# Expression of CCRL2 Inhibits Tumor Growth by Concentrating Chemerin and Inhibiting Neoangiogenesis

**DOI:** 10.3390/cancers13195000

**Published:** 2021-10-05

**Authors:** Diana Al Delbany, Virginie Robert, Ingrid Dubois-Vedrenne, Annalisa Del Prete, Maxime Vernimmen, Ayoub Radi, Anne Lefort, Frédérick Libert, Valérie Wittamer, Silvano Sozzani, Marc Parmentier

**Affiliations:** 1 I.R.I.B.H.M and Welbio, Campus Erasme, Université Libre de Bruxelles, 808 Route de Lennik, B-1070 Brussels, Belgium; delbanidiana@outlook.com (D.A.D.); virginie.gavioli@evotec.com (V.R.); gridou24@hotmail.fr (I.D.-V.); maximevernimmen@hotmail.com (M.V.); ayoub.radi@outlook.com (A.R.); anne.lefort@ulb.be (A.L.); frederick.libert@ulb.be (F.L.); valerie.wittamer@ulb.be (V.W.); 2 Evotec SAS, 195 Route d’Espagne, 31036 Toulouse, France; 3 Institute for Medical Immunology, Université Libre de Bruxelles, Rue Adrienne Bolland 8, 6041 Gosselies, Belgium; 4 Department of Molecular and Translational Medicine, University of Brescia, Viale Europa 11, 25123 Brescia, Italy; annalisa.delprete@unibs.it; 5 Humanitas Clinical and Research Center—IRCCS, Via Manzoni 56, 20089 Rozzano, Italy; 6 Laboratory Affiliated to Istituto Pasteur Italia-Fondazione Cenci Bolognetti, Department of Molecular Medicine, Sapienza University of Rome, 00185 Rome, Italy; silvano.sozzani@uniroma1.it; 7 IRCCS Neuromed, 86077 Pozzilli, Italy

**Keywords:** chemerin, ChemR23, CMKLR1, *Rarres2*, tumor angiogenesis

## Abstract

**Simple Summary:**

Chemerin is a multifunctional protein regulating inflammation, immune responses, and metabolism. It was also shown to display anti-tumoral properties in various cancer models. CMKLR1 is the main functional receptor of chemerin. C-C motif chemokine receptor-like 2 (CCRL2) is another receptor binding chemerin with high affinity but failing to signal through any known signaling pathway. CCRL2 is strongly upregulated by inflammatory signals and was shown to regulate inflammatory reactions in diverse pathological conditions. Expression of CCRL2 was described in many types of human tumors such as melanoma, neuroblastoma, prostate, breast, and gastric cancer. However, its functional role in cancer has not been studied much so far. We investigate in this study how CCRL2 expression can influence the distribution of chemerin and thereby its biological activity in different tumoral contexts.

**Abstract:**

CCRL2 belongs to the G protein-coupled receptor family and is one of the three chemerin receptors. It is considered as a non-signaling receptor, presenting chemerin to cells expressing the functional chemerin receptor ChemR23/CMKLR1 and possibly GPR1. In the present work, we investigate the role played by CCRL2 in mouse cancer models. Loss of function of *Ccrl2* accelerated the development of papillomas in a chemical model of skin carcinogenesis (DMBA/TPA), whereas the growth of B16 and LLC tumor cell grafts was delayed. Delayed tumor growth was also observed when B16 and LLC cells overexpress CCRL2, while knockout of *Ccrl2* in tumor cells reversed the consequences of *Ccrl2* knockout in the host. The phenotypes associated with CCRL2 gain or loss of function were largely abrogated by knocking out the chemerin or *Cmklr1* genes. Cells harboring CCRL2 could concentrate bioactive chemerin and promote the activation of CMKLR1-expressing cells. A reduction of neoangiogenesis was observed in tumor grafts expressing CCRL2, mimicking the phenotype of chemerin-expressing tumors. This study demonstrates that CCRL2 shares functional similarities with the family of atypical chemokine receptors (ACKRs). Its expression by tumor cells can significantly tune the effects of the chemerin/CMKLR1 system and act as a negative regulator of tumorigenesis.

## 1. Introduction

Cancer is the result of a multi-step process requiring the accumulation of mutations in different oncogenes and tumor suppressor genes in the progeny of a single cell [1]. The tumor microenvironment is well known to influence many aspects of cancer development. Various leukocyte populations contribute largely to this microenvironment, and leukocyte chemoattractant molecules, including chemokines, play therefore key roles in cancer progression, more particularly by tuning cancer-related inflammation [2]. The expression of chemoattractant molecules and their receptors is partly caused by genetic events that contribute to neoplastic transformation and play an important role in chronic inflammatory conditions that predispose to cancer. Components of the leukocyte trafficking system affect multiple aspects of tumor progression, including leukocyte recruitment, tumor cell proliferation and survival, neoangiogenesis, epithelial to mesenchymal transition, local invasion, and metastasis.

Chemerin, encoded by the retinoic acid receptor responder 2 (*RARRES2*) gene, also known as tazarotene-induced gene 2 (*TIG2*), is a small secreted protein endowed with chemotactic activity for monocytes/macrophages, myeloid and plasmacytoid dendritic cells, and NK cells [3]. Chemerin binds to three G protein-coupled receptors (GPCR): CMKLR1 (also known as ChemR23 or chemerin_1_), GPR1 (or chemerin_2_), and CCRL2 [4]. A large number of studies have investigated the role of chemerin in tumor biology and the potential value of chemerin as a diagnostic or prognostic biomarker in various cancer types [5,6]. Downregulation of chemerin expression was described in many cancers, including non-small-cell lung carcinoma (NSCLC) [7], squamous cell carcinoma of the skin [6], and melanoma [8], and such downregulation was often associated with poor clinical outcomes. In line with this observation, chemerin expression by cancer cells was shown to delay tumor growth and progression in various mouse models. Immune cell recruitment to the tumor microenvironment, direct effects on cancer cells, and a reduction of tumoral angiogenesis were proposed as mechanisms [7,9,10].

Atypical chemokine receptors (ACKR) have emerged as new regulators of the chemokine system, given their capacity to control chemokine bioavailability and the signaling of functional receptors [11,12]. ACKRs were shown to play essential roles in tumor biology in nearly every key step of progression from tumor initiation to metastasis, including cancer cell proliferation, the recruitment of tumor-associated leukocytes, tumoral angiogenesis, adherence to endothelium and extravasation, and defense against host immune responses [13]. ACKRs influence the behavior of malignant cells as well as of various stromal cells. ACKRs can function in a cell-autonomous manner, affecting the response of the cells in which they are expressed, or indirectly by influencing chemokine receptor signaling in other cells [13]. CCRL2 is structurally related to chemokine receptors CCR1 to 5, but identified as an atypical receptor for chemerin [14,15]. Both human and mouse CCRL2 lack the consensus “DRYLAIV” motif involved in the coupling to heterotrimeric G proteins, which led to the hypothesis that CCRL2 does not function as a classical chemoattractant receptor. CCRL2 binds chemerin with high affinity and shows a low level of constitutive endocytosis that is not enhanced following chemerin binding. CCRL2 is therefore considered as a chemerin binding site, controlling the bioavailability of the protein by increasing its local concentration and presenting it to other cells expressing CMKLR1, the main functional receptor of chemerin [14]. Different cell types express CCRL2 in humans and mice, including macrophages, neutrophils, dendritic cells, microglia, and endothelial cells [16]. CCRL2 expression by tumor cells was also described in several human cancer types, including glioblastoma [17], breast [18], and colorectal cancer [19]. However, the functional role of CCRL2 in cancer has been investigated in few studies so far. A recent study showed that CCRL2 played an anti-tumoral role by contributing to the recruitment of NK cells to tumors in a mouse model of lung cancer [20].

In the present work, we investigate the impact of CCRL2 expression by the host and tumor cells on the chemerin-CMKLR1 axis and its consequences on tumor progression. For this purpose, we used a chemical model of skin carcinogenesis and tumoral cell lines overexpressing or knocked out for *Ccrl2*, as well as various mouse genetic models affecting elements of the chemerin system. We show that expression of CCRL2 by tumor cells enhances the local activity of chemerin, which inhibits neoangiogenesis and results in a reduction of tumor growth and progression.

## 2. Materials and Methods

### 2.1. Mice

C57BL/6J and NOD/SCID mice were purchased from Janvier. The *Ccrl2* [21] and *Cmklr1* [22] knockout mice were described previously. The *Gpr1* knockout line (B6NDen; B6N-*Gpr1*^tm1a(EUCOMM)Hmgu^/Ibcm) was obtained from the EMMA consortium and the chemerin knockout line (C57BL/6N-*Rarres2^tm1(KOMP)Vlcg^*/MbpMmucd) from the Mutant Mouse Resource and Research Center at the University of California at Davis. Mice expressing bioactive chemerin under control of the keratin K5 promoter (K5-chemerin) were described and characterized elsewhere [23]. All strains were backcrossed into the C57BL/6J background for more than 20 generations. Mice were maintained in a specific pathogen-free environment and, except otherwise stated, used between 6 and 12 weeks of age. All animal experiments were conducted following European guidelines and local regulations and approved by the ethics committee (Commission d’Ethique du Bien-Etre Animal, CEBEA) of the ULB Medical School. All efforts were made to minimize suffering.

### 2.2. Cell Lines

Murine B16-F0 melanoma and Lewis lung carcinoma (LLC) cell lines were purchased from the American Type Culture Collection (ATCC). Cells were grown in RPMI-1640 (Life Technologies, Merelbelke Belgium) supplemented with 10% fetal bovine serum (Gibco, Paisley, UK), 1% sodium pyruvate (Gibco), 100 U/mL penicillin, and 100 µg/mL streptomycin (Invitrogen, Carlsbad, CA, USA). The cells were cultured at 37 °C in a humidified atmosphere containing 5% CO_2_. The various clones were regularly tested negative for mycoplasma infection.

### 2.3. Knockout of Ccrl2 in Tumoral Cell Lines

LLC and B16 clones knocked out for *Ccrl2* were generated using an approach that combines a mutant Cas9 nickase and paired CRISPR guide RNAs (sgRNA) targeting the region immediately following the AUG start codon in the second and only coding exon of the *Ccrl2* gene. The sgRNAs, designed using the CRISPR design tool available on the Zhang Lab website (http://crispr.mit.edu) (accessed on 14 October 2016) were 5′-GGATTAGAATCTGCTGATGGACC-3′ (Guide A) and 5′-GGCCTTGAACCAGGCCGGGTGAC-3′ (Guide B) and were cloned into the pSpCas9n (BB)-2A-GFP (PX461) vector (Addgene, Teddington, UK). Following sequencing, the plasmids were cotransfected in LLC and B16 cells using Lipofectamine 2000 (ThermoFisher, Waltham, MA, USA) according to the manufacturer’s instructions. Three days after transfection, single GFP-positive cells were sorted into 96-well plates using a FACSAria cell sorter (BD Biosciences), and the clonal growth was controlled for a week after sorting. The clones were screened by PCR using 5′-TGTCGGATGGAGGGGAATCA-3′ as the forward primer and 5′-CCAAGATAAACACCGCCAGC-3′ as the reverse primer flanking the sgRNA target sites, and a DNA heteroduplex mobility assay on polyacrylamide gels was used to detect small deletions or insertions (1 to 4 bp). The presence of frameshifts and premature stop codons in the two alleles was characterized by cloning the PCR products and sequencing a minimum of 5 clones per cell line.

### 2.4. Overexpression of CCRL2 in Tumoral Cell Lines

LLC and B16 stably overexpressing CCRL2 were generated by transfecting cells with a bicistronic expression plasmid (pCDNeo) containing a codon-optimized version of the mouse *Ccrl2* cDNA and the neomycin (G418) resistance gene driven by an EF-1α promoter. Cells were seeded into 24-well plates containing serum-free medium and transfected using the X-tremeGENE Transfection reagent (Roche, Mannheim, Germany) according to the manufacturer’s instructions. Two days after transfection, the cells were re-seeded onto Petri dishes in a medium containing 800 μg/mL G418 (Invivogen), and individual G418-resistant clones were selected. The level of CCRL2 expression by B16-CCRL2 and LLC-CCRL2 clones was determined by PCR using 5′-ACGAGCCCAGAATGGAGAGA-3′ as the forward primer and 5′-GCTTGTGCAGGTCGTACTGT-3′ as the reverse primer (specific to the codon-optimized *Ccrl2* sequence), and by FACS analysis using a phycoerythrin (PE)-conjugated anti-mouse CCRL2 monoclonal antibody (clone BZ2E3, BD Pharmingen, San Diego, CA, USA).

### 2.5. Proliferation Assays

The proliferation rate of the various B16 and LLC clones was determined by two procedures. Cells were seeded in 96-well plates (1000 cells/well in 100 μL of growth medium) and cultured for 1 to 4 days. Every day, the medium was removed from part of the wells, and the cells were incubated for 4 h at 37 °C with 10 µL of a 5 mg/mL 3-(4,5-dimethylthiazol-2-yl)-2,5 diphenyl tetrazolium bromide (MTT, Sigma–Aldrich, Overijse, Belgium) solution. DMSO (100 µL) was added and the wells gently shaken until dissolution of the formazan crystals. The absorbance at 570 nm was recorded in a microplate reader (BioRad, Temse, Belgium), and cell proliferation was inferred by dividing the absorbance at day X by the absorbance at day 1. The experiment was performed three times with sextuplicate wells.

B16 and LLC cells were also seeded in 6-well plates at densities of respectively 0.25 and 0.3 × 10^6^ cells per well and cultured for 3 days. The cells were collected every day from part of the wells and counted using an EVE automatic cell counter (NanoEnTek). Cell proliferation was determined by dividing the cell number at day X by the number of cells seeded at day 0. The doubling time of the clones was also determined regularly as described previously [24]. Briefly, after harvesting the cells, 10 μL of the suspension was stained with trypan blue, and live cells were counted in the EVE counter. The ratio was calculated since the previous passage and the doubling time measured twice a week for each cell line.

### 2.6. Chemerin Presentation Assay

The ability of cells expressing CCRL2 to bind recombinant mouse chemerin and present it to cells expressing CMKLR1 was assessed using an aequorin-based calcium mobilization assay [25,26,27]. CHO-K1 cells co-expressing mouse CMKLR1, apoaequorin and Gα16, and control CHO-K1 cells expressing only apoaequorin and Gα16, were collected from plates with phosphate-buffered saline (PBS) supplemented with 5 mM EDTA, pelleted, resuspended at a density of 5 × 10^6^ cells/mL in aequorin buffer (DMEM/Ham’s F12 containing 0.5% BSA), and incubated with 5 µM coelenterazine H (Promega) for 4 h at room temperature under gentle agitation in the dark. Cells were then diluted 10-fold and incubated for one hour. B16, B16-CCRL2, and B16-Crispr*^Ccrl2^* cells were grown to confluence in 96-well microplates and starved for 24 h in a serum-free medium. The next day, the cells were incubated at 37 °C for 2 h with 5 nM recombinant mouse chemerin (R&D Systems) in PBS containing 0.1% BSA. The cells were then washed with PBS/BSA to remove unbound chemerin, and 50 µL of aequorin buffer were added to the wells. CHO cells expressing or not CMKLR1 (25,000 cells in 50 µL) were added to wells containing B16, B16-CCRL2, or B16-Cripr*^Ccrl2^* cells preincubated with chemerin, and luminescence was recorded for 20 s in a Packard luminometer. Results (as luminescence units) were normalized to the response to 10 µM ATP.

### 2.7. GFP-Chemerin Binding Assay

A recombinant baculovirus containing a GFP-chemerin fusion was constructed by the Bac-to-Bac system (Invitrogen). The GFP-chemerin fusion was cloned into the pFastBac vector (Invitrogen) between restriction sites *Nde*I and *Not*I, and the plasmid transferred into DH10Bac E. coli cells in order to generate the recombinant bacmid. SF9 cells were transfected with the bacmid to generate the viral stock. For the GFP-chemerin fusion protein production, SF9 cells were infected with the viral stock, and the supernatant was collected 72 h later. The fusion protein was purified on a HiTrap heparin column (Cytiva 17-0406-01), and the concentration of recombinant protein was determined by ELISA and Western blotting, using recombinant chemerin as a reference control. Tumor cells were grown on polylysine-treated 24-well glass bottom sensoPlates (Greiner, 662892, Kremsmünster, Austria) to 60–80% confluence. Cells were incubated in serum-free media for 24 h, then with 10 nM of GFP-chemerin for 2 h at 37 °C. The ligand was removed, and the cells were fixed for 20 min with 4% paraformaldehyde and washed three times with PBS. They were further incubated overnight at 4 °C with a rabbit anti-GFP antibody (Abcam, Ab6550), then for 2 h in the dark at room temperature with an Alexa Fluor 488-conjugated goat anti-rabbit IgG (1:500, Invitrogen). Nuclei were stained with Hoechst 33,342 (1:4000, Life Technologies). Images were acquired using a Zeiss AxoImager Z1 microscope (Carl Zeiss, Jena, Germany) and analyzed with the ImageJ software.

### 2.8. Tumor Models

B16-F0 or LLC cells (10^6^) were grafted subcutaneously into the back of syngeneic C57BL/6J mice. The size of the resulting tumors was monitored every other day from day 3 with a caliper, and the tumor volume was estimated by the formula V = (length × width^2^)/2. At the end of the experiment, mice were killed by cervical dislocation, and tumors were collected for further analysis.

The DMBA/TPA two-stage chemical carcinogenesis model was performed as previously described [28], using eight-week-old mice of the C57BL/6J background. Mice were treated during the first and seventh week with 9,10-dimethyl-1,2-benzanthracene (DMBA, Sigma, 50 µg in 200 µL acetone) applied on shaved skin three times at two-day intervals, and with 12-O-tetradecanoyl phorbol-13-acetate (TPA, Sigma, 4 μg in 200 μL acetone), applied twice a week from weeks 2 to 6 and from week 8 onwards. The number and size of tumors were recorded every other week.

### 2.9. Flow Cytometry Analysis

Tumors were cut into small fragments (about 1 mm^3^) and digested for 1 h 30 min at 37 °C on a rocking plate in HBSS medium containing 5% fetal bovine serum, 1 mg/mL collagenase D (Roche), and 200 U/mL DNase I (Roche). After addition of 5 mM EDTA to block collagenase D activity, the cell suspension was rinsed with PBS and tissue debris eliminated by passing through a 70-μm nylon mesh. Single-cell suspensions were incubated for 20 min at 4 °C with anti-CD16/CD32 Fc Block (eBioscience, San Diego, CA, USA) in PBS containing 1% FCS, 1 mM EDTA, and 0.1% NaN3 (FACS buffer) and stained for 30 min at 4 °C with a mixture of antibodies in FACS buffer. Antibodies recognizing CD45 (47-0451 and 17-0451-83), NK1.1 (12-5941-82), F4/80 (BM8), and CD3 (17-0032-82) were from eBioscience, and CD11b (550993 and 553311), CD11c (550261), Gr1 (552093), and B220 (RA3-6B2) from BD Pharmingen. Tumoral cell lines were also stained with an anti-mouse CCRL2 (clone BZ2E3, BD Pharmingen). Flow cytometry analysis was performed on an LSRFortessa instrument (BD Biosciences, Franklin Lakes, NJ, USA) and analyzed using the FlowJo software.

### 2.10. Histological Procedures

Tissues were embedded in OCT (Tissue Tek, Sakura, Berchem, d sectioned at 8 μm using a Leica cryostat. Sections were post-fixed in acetone for 10 min at 4 °C. For immunofluorescence analysis, the sections were blocked with 1% rat serum, incubated overnight at 4 °C with PE-conjugated mouse anti-CD31 and APC-conjugated mouse anti-αSMA (eBioscience), nuclei were stained with Hoechst 33,342 (1:4000, Life Technologies), and slides were mounted in DAKO mounting medium supplemented with 2.5% 4-diazabicyclo [2.2.2] octane (DABCO, Sigma). Images were acquired using a Zeiss Axio Zoom V16 microscope (Carl Zeiss) and analyzed with the ImageJ software.

### 2.11. RT-qPCR

Tumors and tumor cell lines were lysed in TRIzol (Life Technologies), and mRNAs were extracted using the RNAeasy Minikit (Qiagen Benelux, Antwerp, Belgium). RNA samples (1 µg) were treated with DNase I (Invitrogen) and transcribed into cDNA using oligo(dT) and SuperscriptIII (Invitrogen). Reactions were performed in a final volume of 20 µL using the Power SYBR Green PCR Master Mix (ThermoFisher) on a 7500 Fast thermocycler (Applied Biosystems, Waltham, MA, USA). Glyceraldehyde-3-phosphate dehydrogenase (GAPDH) was used as reference. Relative mRNA levels were calculated according to formula 2^−ΔC^_T_, in which ΔC_T_ = C_T_ (target gene) -C_T_ (GAPDH). The primers used were 5′-AAGCTCCAGCAGACCAACTG-3′ (forward) and 5′-TTTACCCTTGGGGTCCATTT-3′ (reverse) for chemerin, 5′-CCATGTGCAAGATCAGCAAC-3′ (forward) and 5′-GCAGGAAGACGCTGGTGTA-3′ (reverse) for CMKLR1, 5′-GAGCAAGGACAGCCTCCGAT-3′ (forward) and 5′-CCACTGTTGTCCAGGTAGTCG-3′ (reverse) for CCRL2, 5′-GCTGCTGCTTATGGGCTTCTC-3′ (forward) and 5′-TCACTGGGCAGTTTCTAGGAG-3′ (reverse) for GPR1 and 5′-AAGGGCTCATGACCACAGTC-3′ (forward) and 5′-CAGGGATGATGTTCTGGGCA-3′ (reverse) for GAPDH.

### 2.12. RNA Sequencing and Transcriptome Analysis

Tumors were lysed in TRIzol, and total RNA extracted with the RNAeasy micro kit (Qiagen) following the manufacturer’s instructions. RNA quality was checked on a Bioanalyzer 2100 (Agilent Technologies, Machelen, Belgium). Indexed cDNA libraries were obtained using the TruSeq RNA sample preparation kit (Illumina, San Diego, CA, USA). The multiplexed libraries (10 pM) were loaded on flow cells, and sequences were produced using a HiSeq PE Cluster Kit v4 and TruSeq SBS Kit v3-HS on a Hiseq 1500 (Illumina). Approximately 35 million paired reads per sample were mapped against the mouse reference genome (GRCm38.p4/mm10, ftp.Ensembl.org) using the STAR software to generate read alignments for each sample. Counts were obtained using HTSeq and differential gene expression was calculated on the Degust website (http://www.vicbioinformatics.com/degust/) (accessed on 13 September 2016) using EdgeR. The data have been deposited in NCBI’s Gene Expression Omnibus and are accessible through GEO Series accession number GSE183914. Gene signatures were analyzed on the Gorilla (http://cbl-gorilla.cs.technion.ac.il) (accessed on 4 October 2016) and GSEA (http://software.broadinstitute.org/gsea/index.jsp) (accessed on 20 October 2016) websites [29,30].

### 2.13. Statistical Analyses

Statistical analyses and data graphing were performed using Prism 6 (GraphPad Software, version 6.01, San Diego, CA, USA). Statistical significance was calculated by the Mann–Whitney test for comparisons between two groups, or by one-way ANOVA followed by Tukey–Kramer multiple comparisons test for more than two groups, as indicated in the figure legends. *p*-values < 0.05 were considered significant.

## 3. Results

### 3.1. CCRL2 Deficiency Accelerates Tumor Progression in a Chemical Model of Skin Carcinogenesis and Delays Tumor Growth in Graft Models

**Figure 1:**
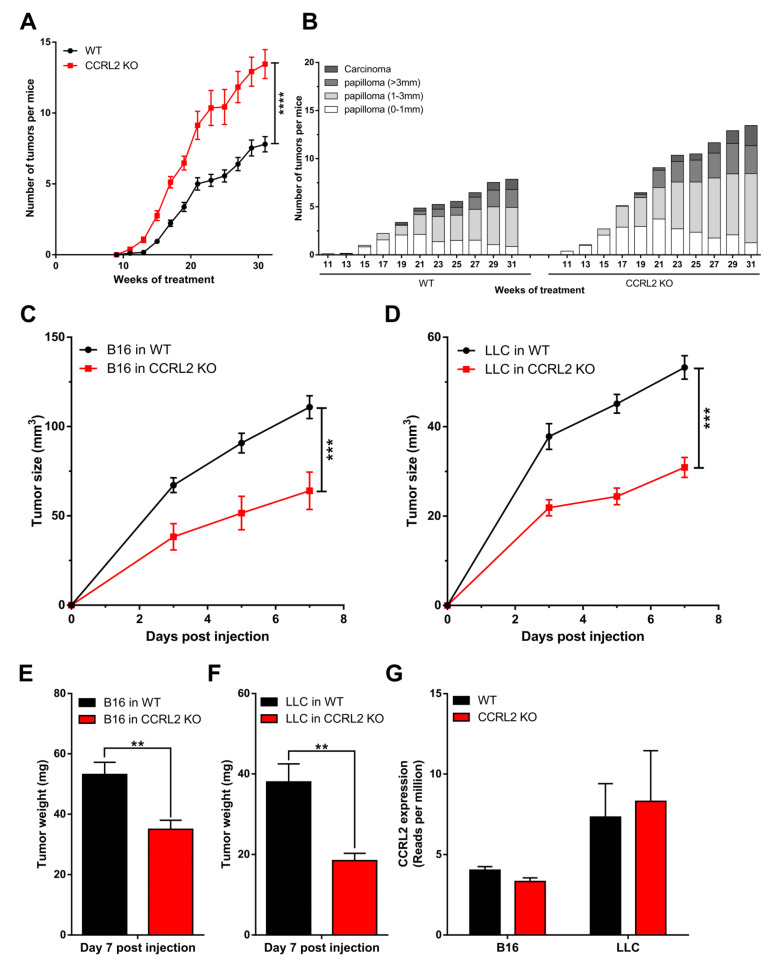
CCRL2 affects tumor growth. (**A,B**) WT and *Ccrl2*-deficient mice were subjected to the DMBA/TPA chemical carcinogenesis model. The number of tumors per mouse (**A**) and the proportion of tumors according to size and stage (**B**) were recorded every other week. (**C**–**F**) WT and *Ccrl2* KO mice were grafted with B16 melanoma cells (**C**) and LLC cells (**D**), and the tumor size was evaluated every other day until day 7. The weight of B16 tumors (**E**) and LLC tumors (**F**) was measured following sacrifice of the mice. Data represent the mean ± SEM, *n* ≥ 5 mice per group, **** *p* < 0.0001, *** *p* < 0.001, ** *p* < 0.01, Mann–Whitney test for all panels. (**G**) LLC and B16 tumor cells express CCRL2 in vivo. The data representing reads per million were extracted from an RNAseq experiment performed on pools of B16 tumors collected 3 days after the graft and LLC tumors collected 5 days after the graft from WT and *Ccrl2* KO mice. For each condition, two pools of 3 tumors were analyzed (mean ± SEM).

*Ccrl2* KO mice and their WT controls were subjected to a two-stage chemical model of skin carcinogenesis (DMBA/TPA) as previously described [23]. *Ccrl2* KO mice developed the first papillomas by week 11 of the treatment, while they appeared only at week 15 in the control group (Figure 1A). *Ccrl2* KO mice also exhibited a larger number of tumors and faster tumor progression compared to control mice (Figure 1A,B). Indeed, the proportion of large papillomas (>3 mm) and carcinomas reached 25.4% in *Ccrl2* KO mice versus 18.9% in control mice by the end of the experiment (Figure 1B). We also investigated the function of CCRL2 in tumor graft models. Following the graft of B16-F0 melanoma or Lewis lung carcinoma cells under the back skin, *Ccrl2* KO mice developed smaller tumors than WT mice (Figure 1C–F). These contrasting results in different tumor models appeared at first puzzling, but it was reasoned that in graft models, the tumor cell lines are not knocked out for *Ccrl2*. It is well known that CCRL2 is upregulated in inflammatory conditions, which are frequently present in the tumor microenvironment, and expression of this receptor by tumor cells was reported in several human cancer types. We therefore tested the presence of *Ccrl2* transcripts by qRT-PCR and confirmed the expression of the receptor in LLC and B16 tumors collected from WT and *Ccrl2* KO mice (data not shown). These results were confirmed in RNAseq data obtained on these tumors (Figure 1G). The expression level was somewhat higher in LLC tumors than in B16 tumors and unaffected by the genotype of the mice (*Ccrl2* KO or WT), demonstrating that the transcripts derive primarily from tumor cells and less from stromal cells of the microenvironment, such as leucocytes or endothelial cells.

### 3.2. The Consequences of Ccrl2 Loss of Function Are Linked to the Chemerin/CMKLR1 System

**Figure 2:**
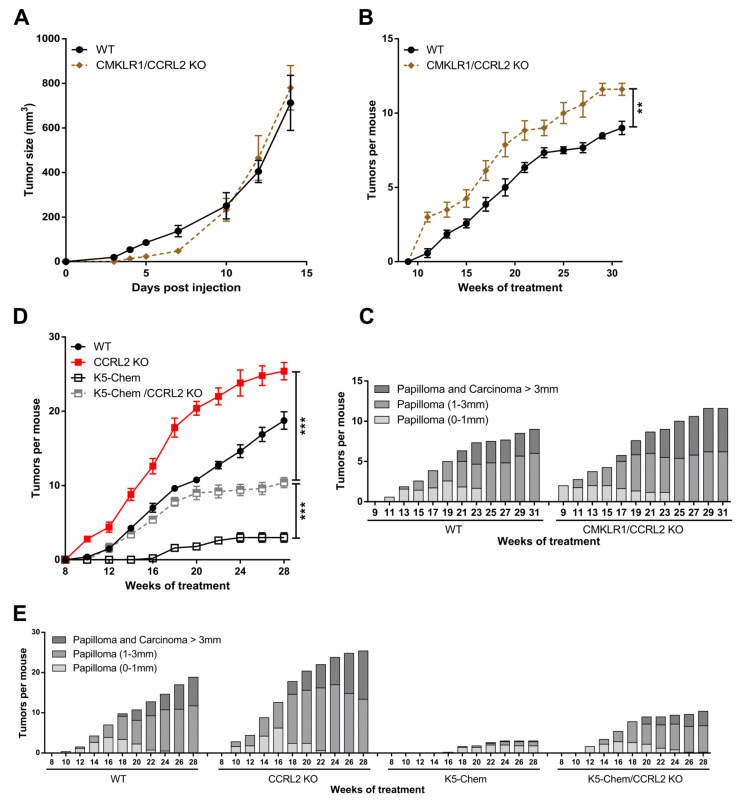
The effects of CCRL2 on tumor growth are mediated by CMKLR1. (**A**) WT and *Cmklr1*/*Ccrl2* double KO mice were grafted with LLC cells, and the tumor size was measured over time. Representative experiment out of three performed (mean ± SEM, *n* ≥ 5 mice per group). (**B**,**C**) WT and *Cmklr1*/*Ccrl2* double KO mice were subjected to the DMBA/TPA chemical carcinogenesis model. The number of tumors per mouse (mean ± SEM) **(B)** and the size and the grade of the tumors (**C**) were recorded. (**D**,**E**) WT, K5-chemerin, *Ccrl2* KO, and K5-chemerin/*Ccrl2* KO mice were subjected to the DMBA/TPA chemical carcinogenesis model. The number of tumors per mouse (mean ± SEM) (**D**) and the proportion of tumors according to size and stage (**E**) were recorded every other week. *** *p* < 0.001, ** *p* < 0.01, Mann–Whitney for panels A and B, one-way ANOVA for panel D. The data are the compilation of three independent experiments with *n* ≥ 5 mice per group in each experiment.

To determine whether CMKLR1 is involved in the effects induced by the knockout of *Ccrl2*, mice deficient for both *Cmklr1* and *Ccrl2* (*Cmklr1*/*Ccrl2* KO) and their WT controls were grafted with LLC and B16 cells. No difference in tumor growth was recorded between the two groups (Figure 2A and not shown), suggesting that the effects of CCRL2 on tumor growth in the tumoral graft models are entirely mediated by the chemerin/CMKLR1 system. In the chemical carcinogenesis model, *Cmklr1*/*Ccrl2* KO mice had a phenotype similar to that observed for *Ccrl2* KO mice, with tumors appearing in a larger number than in WT mice (Figure 2B,C), although the difference was less important. In this model, it seems that part of the effect of *Ccrl2* loss of function is dependent on CMKLR1, but that the phenotype might also be mediated either by the third chemerin receptor GPR1 or by an unidentified chemerin-independent mechanism.

Using a transgenic mouse model overexpressing bioactive chemerin under the control of the keratin K5 promoter, we have shown recently that expression of chemerin by keratinocytes inhibits tumor development in the DMBA/TPA model, and this effect is mediated by the main receptor of chemerin, CMKLR1 [23]. To determine whether CCRL2 is involved in the protective effects of chemerin, we used mice overexpressing chemerin and deficient for CCRL2 (K5-chemerin/*Ccrl2* KO) and subjected them to the DMBA/TPA model. As previously reported, K5-chemerin mice developed a lower number of tumors than their WT controls, and *Ccrl2* knockout increased the number of tumors (Figure 2D). K5-chemerin/*Ccrl2* KO mice presented an intermediate phenotype. The number of tumors developed by K5-chemerin/*Ccrl2* KO mice was relatively similar to that of control mice during the first weeks and stabilized from around week 20 (Figure 2E). These data suggest that CCRL2 contributes to the anti-tumoral effects of chemerin in the overexpression model, and on the other hand, that chemerin overexpression neutralizes the consequences of *Ccrl2* loss of function.

### 3.3. Knocking out Ccrl2 in Tumor Cells Restores the Growth of Tumor Grafts in Ccrl2 KO Mice

**Figure 3:**
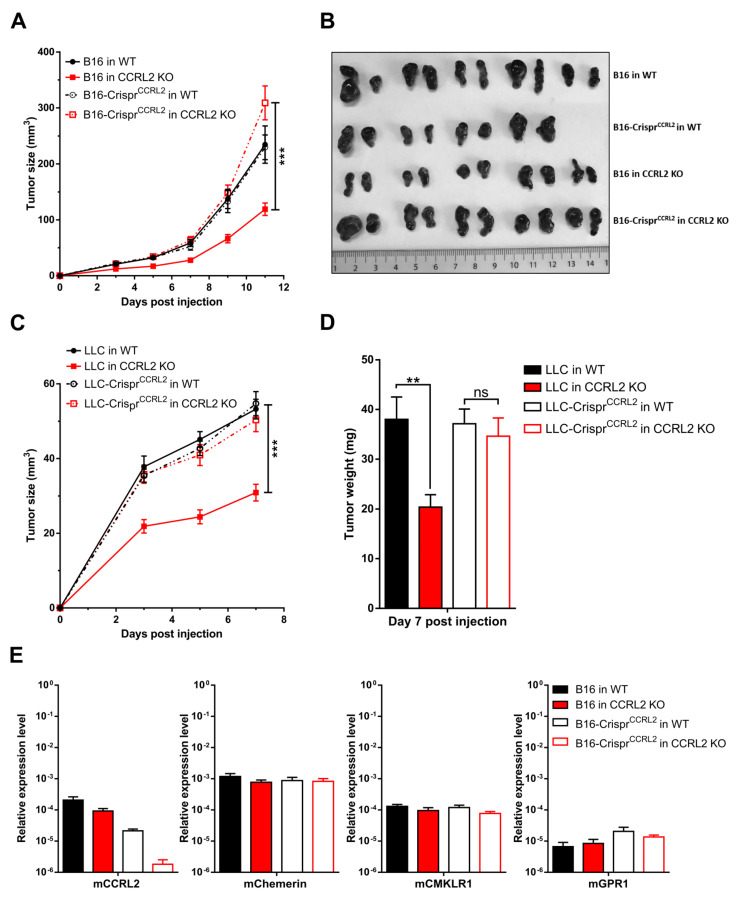
*Ccrl2* knockout in tumor cells abolishes the consequences of *Ccrl2* loss of function in the host. B16 and LLC clones knocked out for *Ccrl2* were generated by the CRISPR/Cas9n technology. (**A**,**B**) B16 cells and a B16 clone knocked out for *Ccrl2* (B16-Crispr*^Ccrl2^*) were grafted to WT and *Ccrl2* KO mice. The size of the tumors was monitored up to day 11 (**A**), and the tumors collected following sacrifice are shown (**B**). (**C**,**D**) LLC cells and an LLC clone knocked out for *Ccrl2* (LLC-Crispr*^Ccrl2^*) were grafted to WT and *Ccrl2* KO mice. The size of the tumors was monitored up to day 7 **(C)**, and the weight of the tumors measured following sacrifice (**D**). (**E**) Chemerin, CMKLR1, CCRL2, and GPR1 transcript levels were evaluated by qRT-PCR in B16 tumors collected on day 11. *** *p* < 0.001, ** *p* < 0.01, one-way ANOVA followed by Tukey–Kramer test for all panels. Data from two independent experiments are shown and expressed as mean ± SEM.

Several studies have reported the expression of CCRL2 by tumor cells [17,19], and we demonstrated that B16 and LLC cells express CCRL2 in vivo. Therefore, we investigated the potential effect of CCRL2 expression by tumor cells on tumor growth. We generated LLC and B16 cell lines knocked out for *Ccrl2* (B16-Crispr*^Ccrl2^* and LLC-Crispr*^Ccrl2^* cells). The approach combined a mutant Cas9 nickase and a pair of guide RNAs (sgRNA) in order to reduce potential off-target mutagenesis events, which are frequent with wild-type Cas9. The presence of mutations in *Ccrl2* alleles was tested in a set of clones by PCR amplification and a DNA heteroduplex mobility assay on polyacrylamide gels, followed by sequencing of the cloned PCR product. Several B16-Crispr*^Ccrl2^* and LLC-Crispr*^Ccrl2^* cell lines baring frameshift deletions in both alleles were selected (Figure S1). B16-Crispr*^Ccrl2^* and LLC-Crispr*^Ccrl2^* cells were grafted subcutaneously into the back of WT and *Ccrl2* KO mice (Figure 3A–D). B16-Crispr*^Ccrl2^* or LLC-Crispr*^Ccrl2^* cells generated tumors of similar size in WT and *Ccrl2* KO mice, while delayed tumor growth was observed in *Ccrl2* KO mice injected with control B16 (Figure 3A,B) or control LLC cells (Figure 3C,D). Similar results were obtained with two independent clones of B16 and LLC cells knocked out for *Ccrl2* (data not shown). We evaluated the expression of chemerin and its receptors by qRT-PCR in B16 tumors of *Ccrl2* KO and WT mice. CCRL2 expression was moderately affected by the knockout of the host gene, but much more by the knockout of the gene in the tumor cell line (Figure 3E). It was down to background levels when knocked out in both the host and the cell line. There was no significant difference in the expression of chemerin, CMKLR1, and GPR1. These data confirm that most *Ccrl2* transcripts in tumors derive from tumor cells and not from the microenvironment, and that this expression by tumor cells is responsible for the delayed tumor growth in *Ccrl2* KO mice.

### 3.4. CCRL2 Overexpression by Tumor Cells Delays Tumor Growth

**Figure 4:**
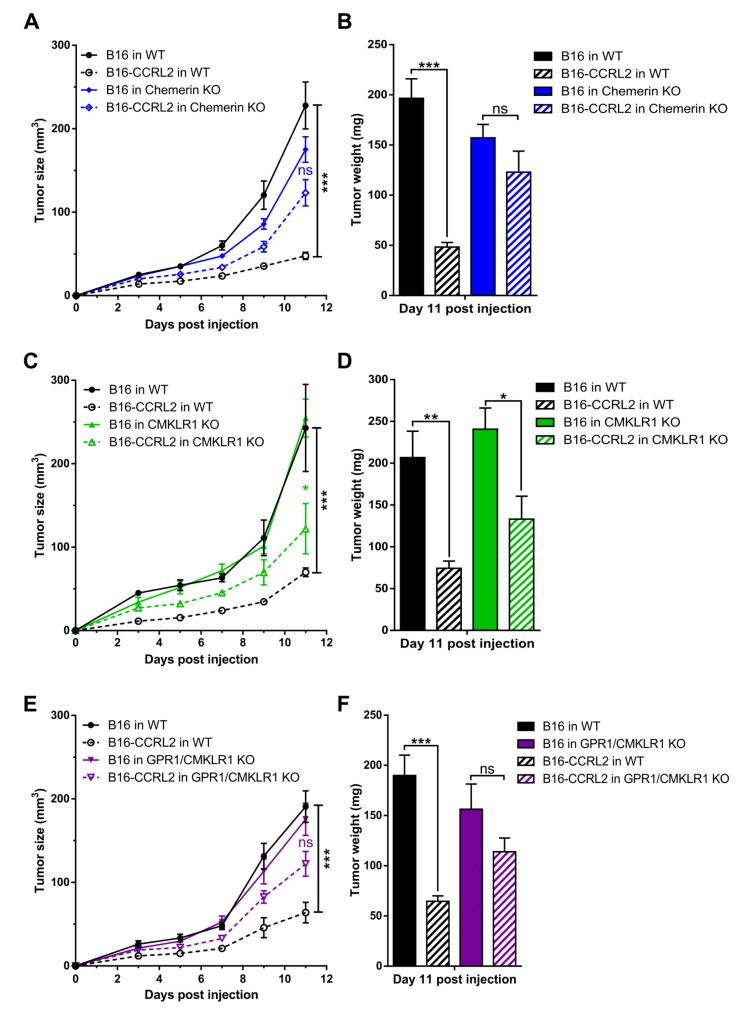
The consequences of CCRL2 overexpression in tumor cells are partially dependent on chemerin and CMKLR1 but independent of GPR1. WT and chemerin KO (**A**), *Cmklr1* KO (**C**), and *Gpr1*/*Cmklr1* KO mice (**E**) were grafted with B16 or B16-CCRL2 cells, and the tumor size measured over time. (**B**,**D**,**F**) The weight of the tumors was measured in each group at day 11 post-graft. The data are the mean ± SEM of three independent experiments with ≥5 mice per group in each experiment. *** *p* < 0.001, ** *p* <0.01, * *p* < 0.05, one-way ANOVA followed by Tukey–Kramer test for all panels.

We next investigated the effect of CCRL2 overexpression by tumor cells on tumor growth in vivo. We generated B16 and LLC clones stably overexpressing a codon-optimized version of mouse *Ccrl2*. Expression of the gene was confirmed by PCR (data not shown) and FACS analysis. B16-CCRL2 and LLC-CCRL2 lines displayed a strong shift (Figure S2) while CCRL2 was undetectable in control cells and clones knocked out for CCRL2 (B16-Crispr*^Ccrl2^* and LLC-Crispr*^Ccrl2^*) in culture. Mice were injected subcutaneously with control B16 or LLC cells or cell lines overexpressing CCRL2. Overexpression of CCRL2 by tumor cells strongly reduced the growth of B16 and LLC tumors in WT mice (Figure 4 and Figure S3). Similar growth delays were observed with different B16 and LLC clones overexpressing CCRL2 (data not shown). We tested whether these effects were dependent on the presence of functional chemerin, *Cmklr1*, and *Gpr1* genes by using mice knocked out for chemerin, *Cmklr1*, *Gpr1*, or *Gpr1*/*Cmklr1*. Control B16 and LLC tumors grew similarly in WT, chemerin KO (Figure 4A,B; Figure S3A,B), *Cmklr1* KO (Figure 4C,D and Figure S3C,D), *Gpr1* KO (Figure S4), and *Gpr1*/*Cmklr1* double KO mice (Figure 4E,F and Figure S3E,F). The consequences of CCRL2 overexpression by tumor cells were partially reversed in chemerin KO, *Cmklr1* KO, and *Gpr1*/*Cmklr1* double KO mice (Figure 4 and Figure S3) but unaffected in *Gpr1* KO mice (Figure S4). These results suggest that the delayed growth observed for cells overexpressing CCRL2 is partially dependent on the chemerin/CMKLR1 system but independent from GPR1.

### 3.5. Overexpression or Knockout of Ccrl2 Do Not Affect Proliferation of B16 and LLC Cells Ex Vivo

Receptors such as chemokine receptors can affect tumor cell proliferation via autocrine mechanisms or by influencing the response of other membrane receptors [31,32], while clonal selection following modification of a cell line may also result in differential growth patterns. Therefore, we tested the proliferation properties of the B16 and LLC clones overexpressing CCRL2 or knocked out for the receptor in culture conditions ex vivo. None of the clones tested displayed a detectable change in their cell proliferation rate in culture, as compared to control cells (Figure S5A,B), nor their doubling time (Figure S5C–F). Therefore, CCRL2 expression by tumor cell lines does not modify the proliferation rate of B16 and LLC cells in vitro, and the selection of clones did not result in significant changes in the growth pattern of the cells. Besides, the data displayed in vivo for a single B16 or LLC clone overexpressing CCRL2 or knocked out for the receptor were reproduced for at least one other clone, with no significant difference in the outcome (data not shown).

### 3.6. The Recruitment of Immune Cells to Tumors Is Not Affected in Ccrl2 KO Mice

**Figure 5:**
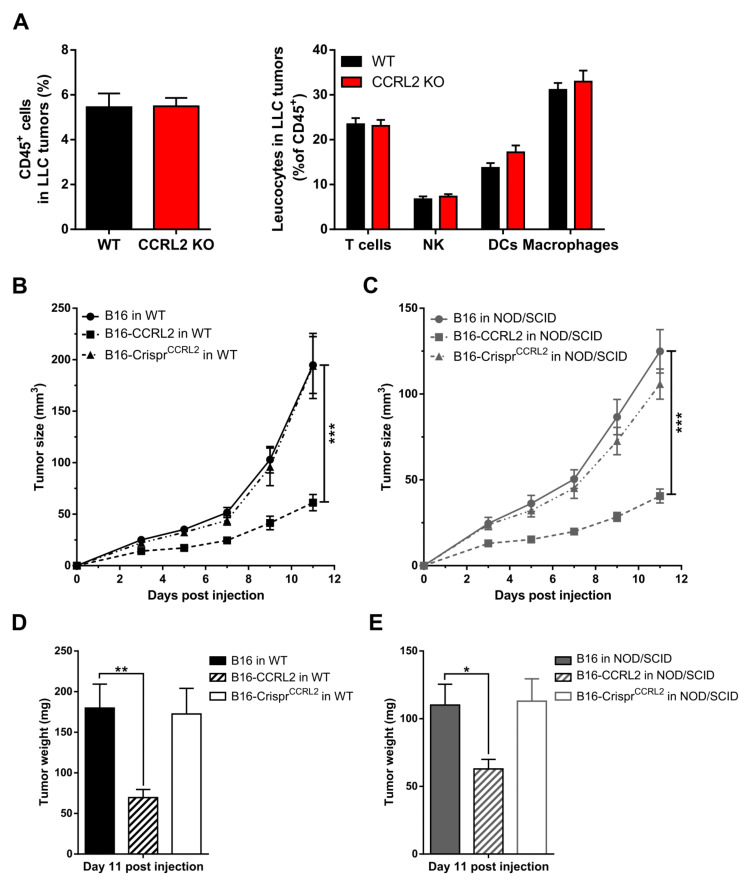
The anti-tumoral effect of CCRL2 is independent of the recruitment of leukocytes. (**A**) LLC cells were grafted to control and *Ccrl2* KO mice. Five days after the graft, the tumors were collected and the cells analyzed by flow cytometry. The percentage of CD45^+^ cells and the proportion of various leukocyte subsets (% of CD45^+^ cells) are represented, including T cells (CD3^+^ NK1.1^−^), NK cells (CD3^−^ NK1.1^+^), DCs (CD11c^+^), and macrophages (CD11b^+^ CD11c^−^ Gr1^−^). Compilation of three independent experiments with 5 mice per condition in each experiment. (**B**–**E**) B16, B16-CCRL2, and B16-Crispr*^Ccrl2^* cells were grafted to WT (**B**) and NOD/SCID mice (**C**), and the tumor size was monitored daily. The weight of the tumors was measured at day 11 post-injection (**D**,**E**). The results (mean ± SEM) are from three independent experiments with *n* = 6 mice per group in each experiment. *** *p* < 0.001, ** *p* < 0.01, * *p* < 0.05, Mann–Whitney for panels A and B, one-way ANOVA followed by Tukey–Kramer test for all other panels.

Various immune cells express CCRL2 [33], including dendritic cells [21] and macrophages [34], and this might affect the trafficking of these cells in response to chemerin [3,14,35]. CCRL2 expression by endothelial cells or other cell types may also contribute to the regulation of leukocyte trafficking [15,20,36] while CCRL2 may modify the functional response of other chemoattractant receptors in cells where the two receptors are co-expressed [37]. Therefore, we examined whether *Ccrl2* deficiency in the host modifies the recruitment of immune cells to the tumor microenvironment. The proportion of leukocytes was assessed by FACS in tumors collected from WT and *Ccrl2* KO mice. No significant changes were observed in the number of CD45^+^ cells, nor in the proportion of different leukocyte subpopulations in LLC tumors at day 5 post-graft (Figure 5A). Similar experiments were also performed on B16 tumors and at different time points with similar outcomes (data not shown). Next, we grafted B16, B16-CCRL2, and B16-Crispr*^Ccrl2^* cells to WT and NOD/SCID immunodeficient mice. Tumors overexpressing CCRL2 were significantly smaller than WT B16 and B16-Crispr*^Ccrl2^* tumors in WT mice (Figure 5B–D) but also in NOD/SCID mice (Figure 5C–E). These results support the concept that, in these tumor graft models, CCRL2 acts on tumor growth independently from the recruitment of leucocyte populations. We also assessed, by qRT-PCR, the expression of several mediators of inflammation, including chemokines (CXCL1, CXCL2, CCL2), cytokines (IL-1β, IL-6, IL-10, IFN-γ), and proteases (MMP9, MMP10) in B16 and LLC tumors collected from WT and *Ccrl2* KO mice. We did not observe any significant differences in the expression of these genes between the two genotypes (data not shown).

### 3.7. CCRL2-Expressing Cells Concentrate Chemerin and Activate CMKLR1^+^ Cells

**Figure 6:**
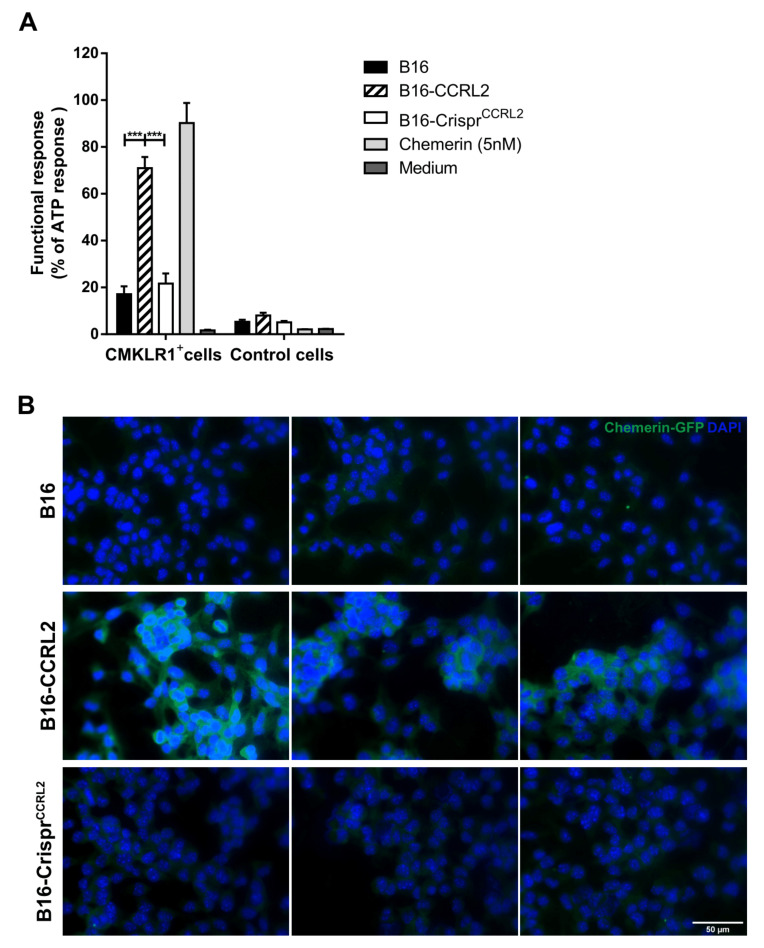
CCRL2 expressed by tumor cells binds and presents chemerin to other cells. (**A**) B16, B16-CCRL2, and B16-Crispr*^Ccrl2^* cells were preincubated with chemerin, washed and then mixed with CHO-K1 cells expressing or not CMKLR1. The functional response of the cells in the aequorin-based calcium mobilization assay was recorded as a luminescent signal and normalized to the response obtained for 10 µM ATP. The data (mean ± SEM) represent the pool of three independent experiments with 3 wells per condition in each experiment. *** *p* < 0.001, one-way ANOVA followed by Tukey–Kramer test. (**B**) B16, B16-CCRL2, and B16-Crispr*^Ccrl2^* cells were incubated for 2 h at 37 °C with 10 nM GFP-chemerin. The cells were washed to remove unbound chemerin, fixed, incubated overnight at 4 °C with an anti-mouse GFP, then 2 h at room temperature with a Cy3-conjugated goat anti-rabbit IgG. The presence of GFP-chemerin was monitored by fluorescent microscopy. The data are representative of 3 individual experiments with similar results. Scale bars = 50 μm.

CCRL2 binds chemerin through its N-terminal domain, leaving the carboxy-terminal peptide critical for cell signaling accessible for an interaction with cells expressing CMKLR1 [14]. CCRL2 acts, therefore, as a chemerin-presenting molecule on barrier cells [36,38]. We hypothesized that CCRL2 (over)expressed by tumor cells might increase the local concentration of chemerin in the tumor and enhance the functional response of CMKLR1-expressing cells in the microenvironment. To test this hypothesis, we compared the ability of WT B16 cells, or clones overexpressing CCRL2 or knocked out for *Ccrl2*, to stimulate CHO-K1 cells expressing CMKLR1 following a pre-incubation with bioactive chemerin. Only cells overexpressing CCRL2 and loaded with chemerin were able to trigger calcium mobilization in CMKLR1-expressing CHO-K1 cells, as measured in an aequorin-based calcium mobilization assay (Figure 6A). WT B16 and B16-Crispr*^Ccrl2^* cells did not generate a specific response in this assay. We next tested whether tumor cells overexpressing CCRL2 could concentrate chemerin on their surface. The various B16 clones, as well as CHO-K1 cells expressing CMKLR1 used as a positive control, were incubated for 2 h at 37 °C in the presence of 10 nM of a GFP-chemerin fusion protein. CMKLR1^+^ CHO-K1 cells bound and internalized GFP-chemerin very efficiently (data not shown), as previously reported [39]. An anti-GFP antibody allowed us to demonstrate efficient binding of GFP-chemerin onto B16-CCRL2 cells (Figure 6B), but no significant internalization of the ligand was observed. In contrast, WT B16 and B16-Crispr*^Ccrl2^* cells ex vivo did not bind detectable levels of GFP-chemerin in this assay (Figure 6B).

### 3.8. CCRL2 Expression by Tumor Cells Impairs Tumor Vascularization

**Figure 7:**
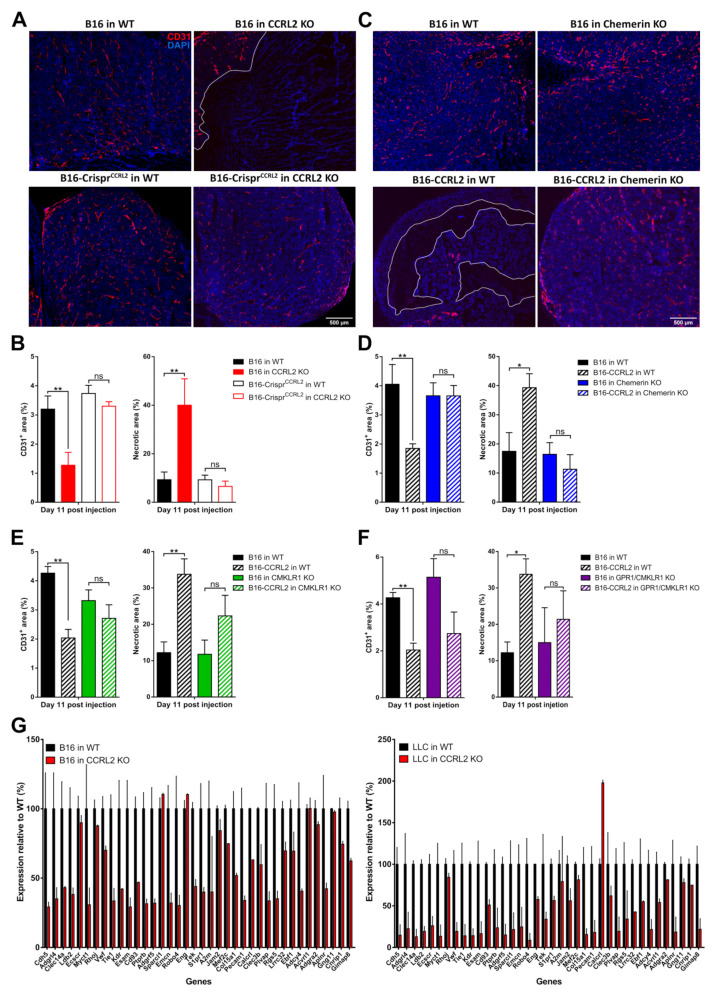
CCRL2 expression by tumor cells regulates neoangiogenesis. (**A**) Immunostaining of CD31 in B16 and B16-Crispr*^Ccrl2^* tumors from WT and *Ccrl2* KO mice collected at day 11 post-injection. (**B**) Relative CD31^+^ and necrosis area in B16 and B16-Crispr*^Ccrl2^* tumors collected from WT and *Ccrl2* KO mice at day 11. (**C**) Immunostaining of CD31 in B16 and B16-CCRL2 tumors from WT and chemerin KO mice collected at day 11 post-injection. (**D**) Relative CD31^+^ and necrosis area in B16 and B16-CCRL2 tumors collected from WT and chemerin KO mice at day 11. (**E**) Relative CD31^+^ and necrosis area in B16 and B16-CCRL2 tumors collected from WT and *Cmklr1* KO mice at day 11. (**F**) Relative CD31^+^ and necrosis area in B16 and B16-CCRL2 tumors collected from WT and *Gpr1*/*Cmklr1* KO mice at day 11. The data (mean ± SEM) represent the pool of three independent experiments with ≥ 5 mice per group in each experiment. ** *p* < 0.01, * *p* < 0.05, one-way ANOVA followed by Tukey–Kramer test for panels B–F. Scale bars = 500 μm. (**G**) Relative expression of the first 38 genes of an angiogenesis signature (described for human cancer types, Masiero et al. 2013) in B16 (left panel) and LLC tumors (right panel) collected from WT and *Ccrl2* KO mice. These data were extracted from RNAseq experiments. For each condition, two pools of 3 tumors, collected respectively at days 3 and 5 for B16 and LLC tumors, were analyzed. The data (mean ± SEM) were normalized relative to the gene expression in WT mice (100%).

Neovascularization is an important component of tumor progression, as it provides oxygen and nutrients to the growing tumor. CCRL2 and CMKLR1 are expressed by endothelial cells, and we demonstrated previously that chemerin expression by tumor cells inhibits tumor angiogenesis, thereby promoting cell death and a growth delay [10]. We therefore investigated the histology of tumors collected from WT and *Ccrl2* KO mice. B16 or LLC tumors grown in *Ccrl2* KO mice presented larger necrotic areas compared to tumors from control mice (Figure 7A,B). Additionally, the relative surface of CD31^+^ staining was significantly smaller in tumors from *Ccrl2* KO mice compared to WT mice (Figure 7B). The knockout of *Ccrl2* in tumor cells restored the vascularization and necrotic area in *Ccrl2* KO mice to a level similar to that observed in WT mice (Figure 7A,B). We next tested whether tumor angiogenesis was also affected by the overexpression of CCRL2 in tumor cells. Tumors overexpressing CCRL2 collected from WT mice showed a significant reduction of the CD31^+^ area and larger necrotic regions compared to control tumors (Figure 7C–F and Figure S6A–D). The consequences of CCRL2 overexpression on neoangiogenesis were mostly reversed in tumors collected from chemerin KO, *Cmklr1* KO, and *Gpr1*/*Cmklr1* KO mice, in which no significant differences in the CD31^+^ and necrosis areas were seen as compared to WT tumors (Figure 7C–F and Figure S6A–D).

We performed RNAseq analyses on B16 and LLC tumors from *Ccrl2* KO and WT mice, isolated at day 5 post-graft. We observed that B16 and LLC tumors from *Ccrl2*-deficient mice displayed a significant downregulation of an angiogenesis signature of 38 genes (Figure 7G) described for a set of human cancer types [40]. However, there were no significant changes in the expression of key pro-angiogenic factors involved in tumoral angiogenesis such as VEGF-A or FGF-2 (data not shown). Altogether, these results show that exclusive or higher CCRL2 expression in tumor cells relative to the host concentrates chemerin locally and inhibits tumor neoangiogenesis, with consequences similar to those resulting from the expression of bioactive chemerin by tumor cells or by the host.

## 4. Discussion

CCRL2 was shown to regulate inflammatory reactions in diverse pathological conditions, including hypersensitivity reactions, arthritis, and experimental autoimmune encephalitis [21,36,37,41], but its role in cancer has been investigated in few studies so far. Increased CCRL2 expression was described in glioma tumors and glioma cell lines [17]. CCRL2 overexpression did not modify the proliferation of the human glioblastoma cell lines U87MG and U373MG in vitro but increased the migration and invasive properties of the cells. The molecular mechanisms and signaling pathways involved were however not determined. High levels of CCRL2 and CCR1 were also found in liver metastases of colorectal carcinoma in rats, but the consequences of CCRL2 expression appeared limited in human primary colorectal carcinoma cells in terms of proliferation, clonogenic capacity, migration, and survival [19]. High expression of CCRL2 was also described in prostate tumors and the PC-3 prostate cancer cell lines [42]. Highly invasive human breast cancer cell lines (MDA-MB-231 and BT-549) were shown to express low levels of CCRL2, and overexpression of the receptor negatively affected the growth of these cells in vitro and in vivo, as well as their chemotactic response to CCL2 and invasive properties [18]. In addition, CCRL2 expression decreased the phosphorylation of p38 MAPK triggered by CCL2 and restored E-cadherin expression. These observations suggest that CCRL2 may act as a tumor suppressor in human breast cancer cells, while displaying pro-tumoral activities in other cancer types. A recent study supported further the antitumoral role of CCRL2 in urethane-induced and mutant Kras models of lung tumor [20]. The delayed tumor progression observed in *Ccrl2* KO mice was associated with a reduction of the recruitment of NK cells, and the involvement of endothelial cells in this phenotype was proposed [20].

In the present study, we analyzed the consequences of *Ccrl2* loss of function in different mouse models of cancer, namely the DMBA/TPA chemical model of skin carcinogenesis and the B16 and LLC graft models. The knockout of *Ccrl2* in mice accelerated the development of tumors in the two-stage skin carcinogenesis model. In contrast, *Ccrl2* deficiency reduced the growth of B16 and LLC tumors in graft models. One significant difference between these models is that *Ccrl2* is not knocked out in the tumor cells in the graft models, while it is absent from all cell types in the chemical carcinogenesis paradigm. Although B16 and LLC do not express high levels of CCRL2 in culture conditions ex vivo, we could demonstrate by qRT-PCR and RNAseq experiments that tumors generated by both cell lines in vivo express significant levels of the receptor, with no significant differences between *Ccrl2* KO and WT mice. This demonstrated unambiguously that the expression of CCRL2 in tumors could be attributed mostly to tumor cells and not to the microenvironment. LLC and B16 cells therefore express CCRL2 *in vivo*, and this expression is likely due to the inflammatory context in the tumor microenvironment, since CCRL2 expression is well known to be strongly upregulated by inflammatory signals in various cell types. This observation opened the possibility that the growth delay observed for grafts in *Ccrl2*-deficient mice might be linked to the exclusive expression of CCRL2 by the tumor cells, resulting in a local concentration of the available bioactive chemerin and enabling chemerin to display its anti-tumoral properties through CMKLR1-expressing cells.

To test this hypothesis, we generated B16 and LLC cell lines in which both *Ccrl2* alleles were inactivated by early frame shifts. Knockout of the *Ccrl2* gene in the tumoral cell lines did not modify the growth of tumors in wild-type mice but abrogated the growth delay that was observed in *Ccrl2* KO mice grafted with unmodified B16 and LLC cells. These observations validated the proposed model, supporting the role of differential CCRL2 expression across different parts of the body for directing the biological effects of chemerin to specific environments. The consequences of *Ccrl2* inactivation in the host were also abrogated by the simultaneous knockout of *Cmklr1*, demonstrating that the phenotype is mediated through the chemerin/CMKLR1 system.

We further investigated the role of CCRL2 on the activity of the chemerin-CMKLR1 axis in tumor progression by testing tumoral cell lines overexpressing CCRL2. Overexpression of CCRL2 by B16 and LLC cells resulted in a slower growth of the tumors in vivo, without affecting the proliferation of cells in culture ex vivo. This effect was largely reversed by the knockout of the chemerin or *Cmklr1* genes in the host, demonstrating the role of the chemerin/CMKLR1 system, while knockout of *Gpr1* did not alter the phenotype. Part of the effects of CCRL2 overexpression remained, however, in *Cmklr1* and chemerin KO mice, suggesting the contribution of a second mechanism that is presently unidentified. This effect might be linked to changes in the functional response of the cells to other membrane receptors, as previously described for atypical chemokine receptors [11].

In order to validate further the model of chemerin presentation by CCRL2, we could demonstrate the binding of a GFP-chemerin fusion protein on the surface of CCRL2-overexpressing tumor cells by immunofluorescence. We also demonstrated that tumor cells overexpressing CCRL2 can display chemerin on their surface in a stable manner, allowing them to trigger functional responses (calcium mobilization in this case) from cells expressing CMKLR1. The results support further that the anti-tumoral effects of CCRL2, when expressed by tumor cells, are mediated by the local concentration of chemerin in the tumor. Such a mechanism was previously described by Monnier et al., showing that CCRL2, expressed by endothelial cells in inflammatory conditions, captured and concentrated chemerin on their surface, contributing thereby to the recruitment of leucocyte populations expressing CMKLR1 and regulating the concentration of the chemoattractant molecule in plasma [15].

We demonstrated previously that the expression of a bioactive form of chemerin by B16 melanoma and Lewis lung carcinoma (LLC) cells delays tumor growth in vivo [10]. A similar tumor growth delay was observed when bioactive chemerin is expressed in the basal keratinocytes of the host mice. These effects did not involve the recruitment of leukocyte populations to the tumors. It was rather found that chemerin prevents efficient angiogenesis in growing tumors, resulting in hypoxia and an increase in necrotic cell death. The anti-tumoral effect of chemerin was entirely mediated through CMKLR1. The phenotype of the tumors overexpressing chemerin and of the tumors overexpressing CCRL2 or grown in *Ccrl2*-deficient mice, as described in the present work, were very similar. We did not observe in any of these situations a significant change in the leucocyte populations recruited to the tumors, and similar growth delays were seen in immunodeficient mice. In addition, in all these settings, we observed a significant decrease of the vascularization of the tumors, and an increase of necrotic and hypoxic areas in these tumors. RNAseq experiments run on B16 and LLC tumors grown in *Ccrl2* KO mice confirmed a 50% reduction of an angiogenesis signature, an observation similar to that made previously on chemerin-expressing tumors [10]. The anti-angiogenic effects of chemerin through a direct effect on endothelial cells has been analyzed elsewhere in vitro and in vivo. We showed that chemerin can promote the regression of neovessels during the development of the vascular retinal network, reduce the efficiency of reperfusion in the hind limb ischemia model [43], and inhibit the angiogenesis process of human umbilical vein endothelial cells (HUVEC) in the bead sprouting assay [10]. These data contrast with previous reports in the literature claiming a pro-angiogenic effect of chemerin [44,45,46], observations that we could not reproduce.

All our data support therefore a model in which a higher expression of CCRL2 in tumor cells affects dramatically the distribution of bioactive chemerin, resulting in its concentration in the area (the tumor) in which CCRL2 expression is the highest. As a result, the cells expressing CMKLR1 are locally stimulated, resulting in a phenotype similar to that observed when chemerin is overexpressed in the tumor. Cell types reported to express CMKLR1 and contributing to the tumor micro-environment include macrophages, myeloid and plasmacytoid dendritic cells, NK cells, endothelial cells, smooth muscle cells, and adipocytes [3,4]. In our tumor graft models, this chemerin bioactivity is essentially targeted toward endothelial cells, resulting in an impairment of the tumor vascularization, leading to hypoxia and cell death by apoptosis and necrosis. A similar mechanism might affect the recruitment or activation of other cells expressing CMKLR1, such as NK cells, although we did not detect such a change in our present models.

In the DMBA/TPA model, we cannot exclude a contribution of leucocyte populations, but the higher number of tumors observed in *Ccrl2* KO mice might be linked also to changes in the distribution of chemerin in the tumor microenvironment. Indeed, the DMBA/TPA model is based on the mutagenic properties of DMBA but requires also the pro-inflammatory and proliferative properties of TPA, as a result of protein kinase C activation. The chronic local skin inflammatory process is expected to generate bioactive chemerin from its precursor, as a result of neutrophil recruitment [47], and to upregulate CCRL2 in many cell populations. It is therefore likely that active chemerin is retained locally by CCRL2 and can exert its anti-tumoral effect as the result of this local concentration. However, in the absence of CCRL2, active chemerin may leak out of the inflamed tissue much more easily, thereby explaining the faster tumor progression in *Ccrl2* KO mice.

## 5. Conclusions

The present work supports the important role of CCRL2 in tuning the activity of the chemerin/CMKLR1 system. The modulation of angiogenesis observed in our tumor grafts complements other effects of CCRL2 on the tumor microenvironment observed in other models. The properties and roles of CCRL2 are very similar to those attributed to atypical chemokine receptors, modulating the anatomical distribution of their respective ligands and thereby modifying the activity of the cognate functional receptors. In this context, CCRL2 appears as a new player in tumorigenesis and angiogenesis, and its expression should be taken into account in the development of potential therapeutic strategies targeting the chemerin/CMKLR1 system.

## Data Availability

The data of RNA sequencing have been deposited in NCBI’s Gene Expression Omnibus and are accessible through GEO Series accession number GSE183914.

## Supplementary Materials

The following are available online at https://www.mdpi.com/article/10.3390/cancers13195000/s1. Figure S1: Knockout of the *Ccrl2* gene in B16 and LLC cells. Figure S2: CCRL2 expression in B16 and LLC tumor cell lines. Figure S3: The consequences of CCRL2 overexpression in LLC tumors are partially dependent on chemerin and CMKLR1 but independent of GPR1. Figure S4: The anti-tumoral effect of CCRL2 is independent of GPR1. Figure S5: Overexpression or knockout of *Ccrl2* do not affect the proliferation rate of B16 and LLC clones in vitro. Figure S6: CCRL2 expression by LLC cells regulates neoangiogenesis.
