# Supplementary material for: Expression of CCRL2 Inhibits Tumor Growth by Concentrating Chemerin and Inhibiting Neoangiogenesis
Source: Cancers (Basel). 2021 Oct 5;13(19):5000. doi: 10.3390/cancers13195000 (PMC8508266; doi:10.3390/cancers13195000)
Supplement: Supplementary file 1 [file cancers-13-05000-s001.zip › cancers-1367786-supplementary.pdf]

Expression of CCRL2 inhibits tumor growth by concentrating chemerin and inhibiting neoangiogenesis

Diana Al Delbany, Virginie Robert, Ingrid Dubois-Vedrenne, Annalisa Del Prete, Maxime Vernimmen, Ayoub Radi, Anne Lefort, Frédérick Libert, Valérie Wittamer, Silvano Sozzani, Marc Parmentier

Mouse CCRL2 gene sequence (part of exon 2):

```
5'..ATGGATAACTACACAGTGGCCCCGGACGATGAATATGATGTCCTAATCTTAGACGACTACCTGGACAACAGTGGGCCGGACCAAGTTCCGGCC..3'
3'..TACCTATTGATGTGTCACCGGGGCCTGCTACTTATACTACAGGATTAGAATCTGCTGATGGACCTGTTGTCACCCGGCCTGGTTCAAGGCCGG..5'
    MetAspAsnTyrThrValAlaProAspAspGluTyrAspValLeuIleLeuAspApsTyrLeuAspAsnSerGlyProAspGlnValProAla
```

Mouse CCRL2 protein sequence

```
WT CCRL2        MDNYTVAPDDEYDVLILDDYLDNSGPDQVPAPEFLSPQQVLQFCCAVFAVGLLDNVLAVFILVKYKGLKNLGNIYFLNLALSNLCFLLPLPFWAHTAAHGESPGNGTCKVLVGLHSS... (361)
```

Truncated proteins

```
B16-Clone12-1   MDNYTVAPDDEYDVLILDD*(20)
B16-Clone12-2   MDNYTVAPDDEYDVLILDDYLDNSGPDHYLDNMDNSVRPLPGQQWAGPQHGQQWPDHYLDNMDNSVRTTTWTTVGRTTTWTTVGQVPAPEFLSPSRCCSSVPPAGAAVLLRGVCGGS
                LGQRAGGVYLGEIQRTQESGEHLLPKPGTFKPVFPASPAVLGPYCSTRGKPWQWDL*(174)

B16-Clone16-1   MDNYTVAPDDEYDVQRTTVQTTWTTVQRTTVQQWAGPSSGPRVPLPPAGAAVLLRGVCGGSLGQRAGGVYLGEIQRTQESGEHLLPKPGTFKPVFPASPAVLGPYCSTRGKPWQWDL*(118)
B16-Clone16-2   MDNYTVAPDDTTVGRTKFRPPSSSPPSRCCSSAARCLRWVSWTTCWRCLSW*(51)

LLC-Clone11-1   MDNYTVAPDDEYDVLILDDYLDNSTIALIDGFSPFDVGSSGPRVPLPPAGAAVLLRGVCGGSLGQRAGGVYLGEIQRTQESGEHLLPKPGTFKPVFPASPAVLGPYCSTRGKPWQWDL*(118)
LLC-Clone11-2   MDNYTVAPDDEYDSSGPRVPLPPAGAAVLLRGVCGGSLGQRAGGVYLGEIQRTQESGEHLLPKPGTFKPVFPASPAVLGPYCSTRGKPWQWDL*(94)

LLC-Clone27-1   MDNYTVAPDDEYDVLILDDYLDNSTIALIDGFSPFDVGSSGPRVPLPPAGAAVLLRGVCGGSLGQRAGGVYLGEIQRTQESGEHLLPKPGTFKPVFPASPAVLGPYCSTRGKPWQWDL*(118)
LLC-Clone27-2   MDNYTVAPDDEYDSSGPRVPLPPAGAAVLLRGVCGGSLGQRAGGVYLGEIQRTQESGEHLLPKPGTFKPVFPASPAVLGPYCSTRGKPWQWDL*(94)
```

Figure S1. Knockout of the *Ccrl2* gene in B16 and LLC cells. The region of the mouse *Ccrl2* gene (second and only coding exon) targeted by the CRISPR/Cas9n strategy is shown. The sequences of the two sgRNAs are shown in blue, the PAM sequences in green, the start codon in red. The first part of the CCRL2 amino acid sequence is shown, as well as the mutant forms encoded by the two alleles in the selected B16 and LLC clones. The modified sequences resulting from frame shifts are gray-shaded and the premature Stop codons (*) in yellow.

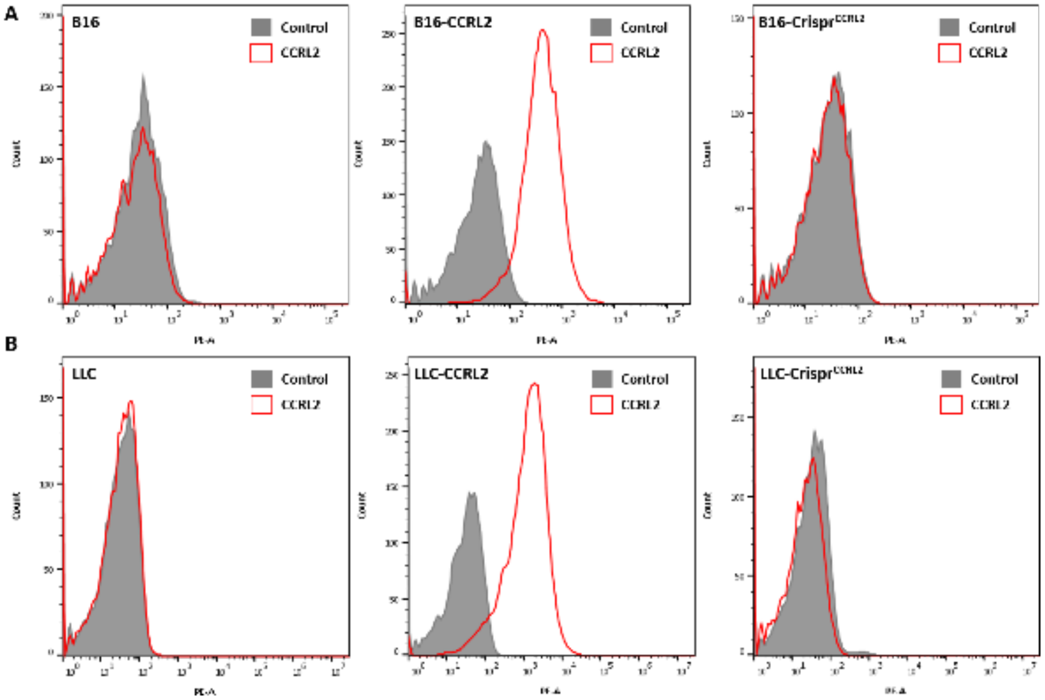

Figure S2. CCRL2 expression in B16 and LLC tumor cell lines. FACS analysis of CCRL2 expression by B16 **(A)** and LLC **(B)** cells and corresponding clones overexpressing CCRL2 or knocked out for the gene by the CRISPR/Cas9 approach. The grey profile is the labeling obtained with an isotype-matched control antibody. The data shown are representative of six independent experiments with similar results.

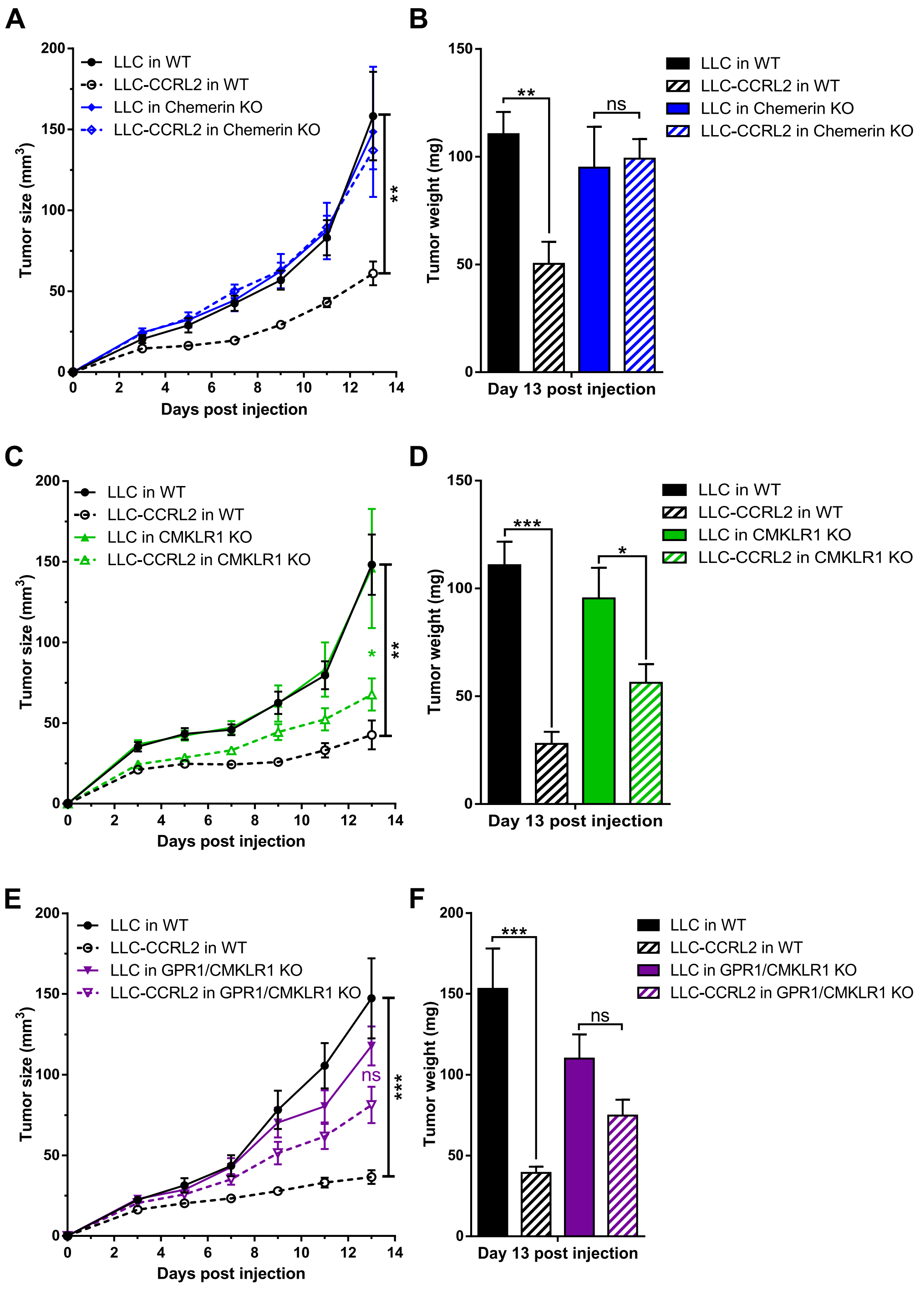

Figure S3. The consequences of CCRL2 overexpression in LLC tumors are partially dependent on chemerin and CMKLR1 but independent of GPR1. WT and chemerin KO **(A)**, *Cmklr1* KO **(C)**, and *Gpr1*/*Cmklr1* KO mice **(E)** were grafted with LLC or LLC-CCRL2 cells, and the tumor size measured over time. **(B, D, F)** The tumor weight of the tumors was measured in each group at day 11 post-graft. The data are the mean ± SEM of three independent experiments with ≥ 5 mice per group in each experiment. ***P<0.001, **P<0.01, *P<0.05, one-way ANOVA followed by Tukey-Kramer test for all panels.

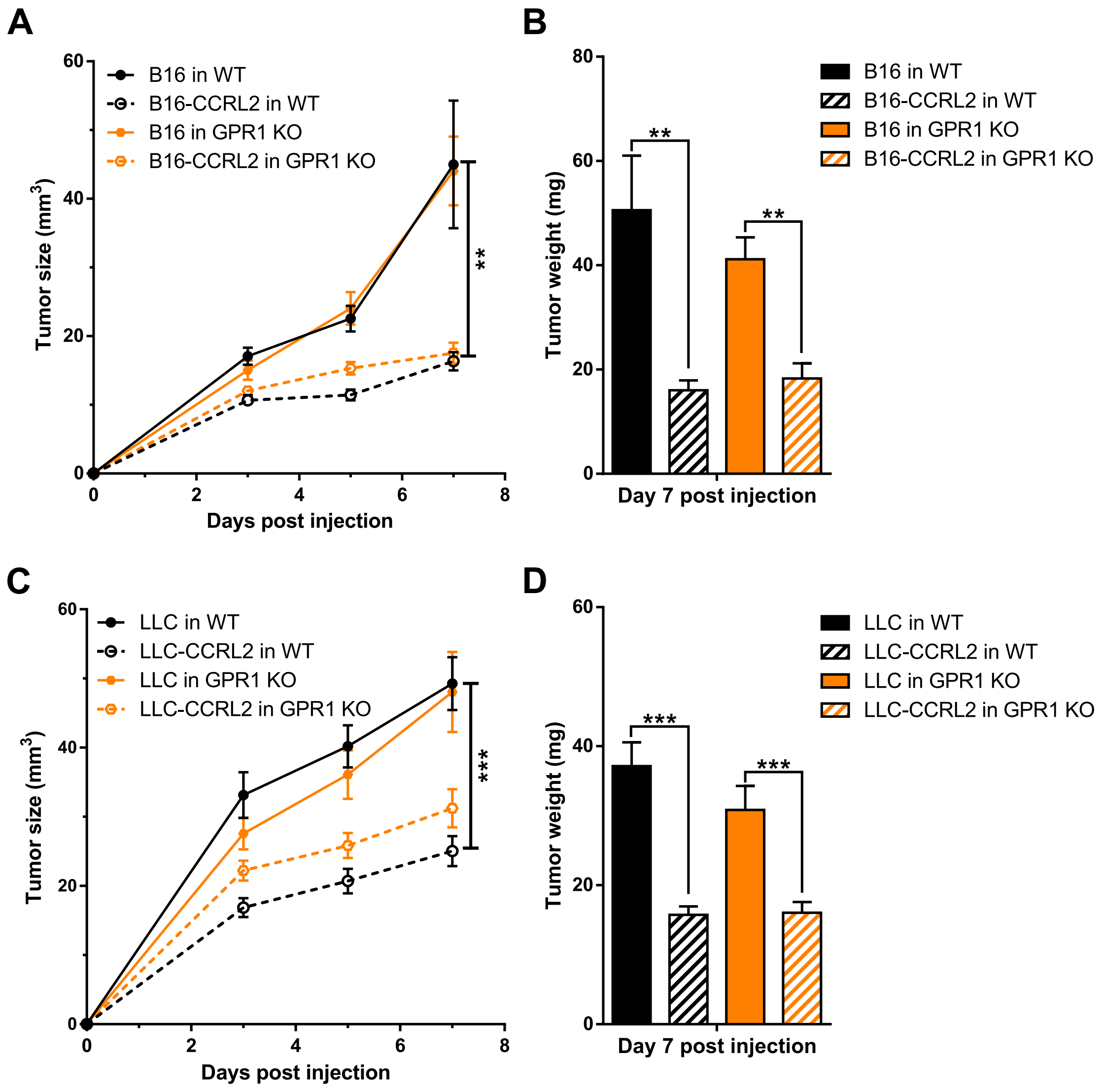

Figure S4. The anti-tumoral effect of CCRL2 is independent of GPR1. B16 or B16-CCRL2 cells **(A),** or LLC and LLC-CCRL2 cells **(C)** were grafted to WT and *Gpr1* KO mice, and the tumor size was measured over time. **(B, D)** The weight of the tumors was measured at day 7 post-graft. The results (mean ± SEM) are the pool of three independent experiments with ≥ 5 mice per group in each experiment. ***P<0.001, **P<0.01, one-way ANOVA followed by Tukey-Kramer test for all panels.

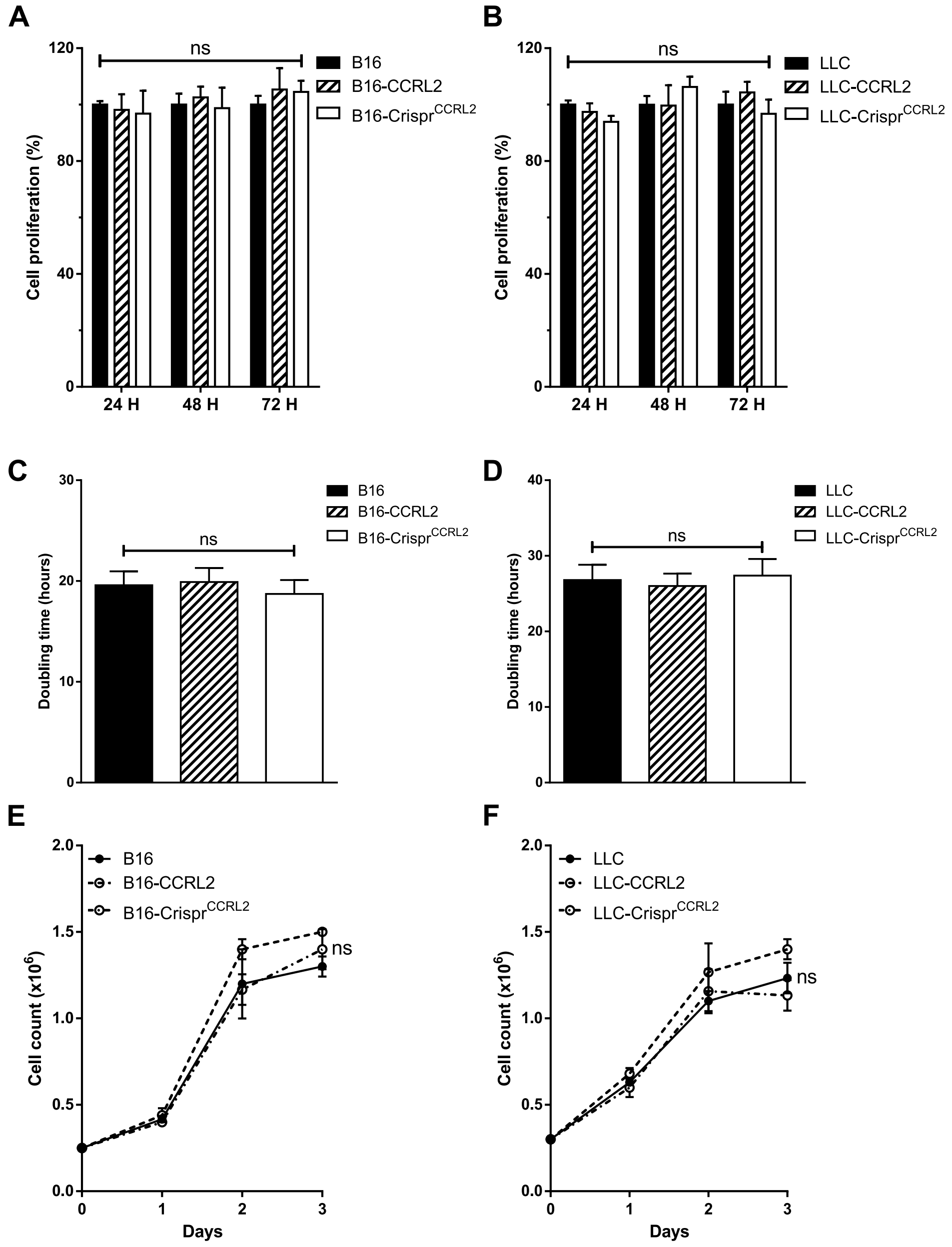

Figure S5. Overexpression or knockout of *Ccrl2* do not affect the proliferation rate of B16 and LLC clones in vitro. The proliferation of selected clones of B16 **(A)** and LLC cells **(B)** overexpressing CCRL2 or invalidated for the gene was compared to that of WT cells by the MTT assay. The values were normalized to the average for WT cells at each time point. The data (mean ± SEM) represent the pool of three independent experiments with six wells per condition in each experiment. **(C, D)** The doubling time of the same clones was calculated by counting the cells at different time points, and using the formula Td = (t2-t1) * log(2)/log(q2/q1), where q1 represents the number of cells at time t1 and q2 represents the number of cells at time t2. **(E, F)** The proliferation rate was also determined by counting the cells on days 1, 2, and 3 and normalizing the values according to the number of cells seeded on day 0. The data (mean ± SEM) are representative of 2 independent experiments with 3 wells per condition. One-way ANOVA followed by Tukey-Kramer test for all panels.

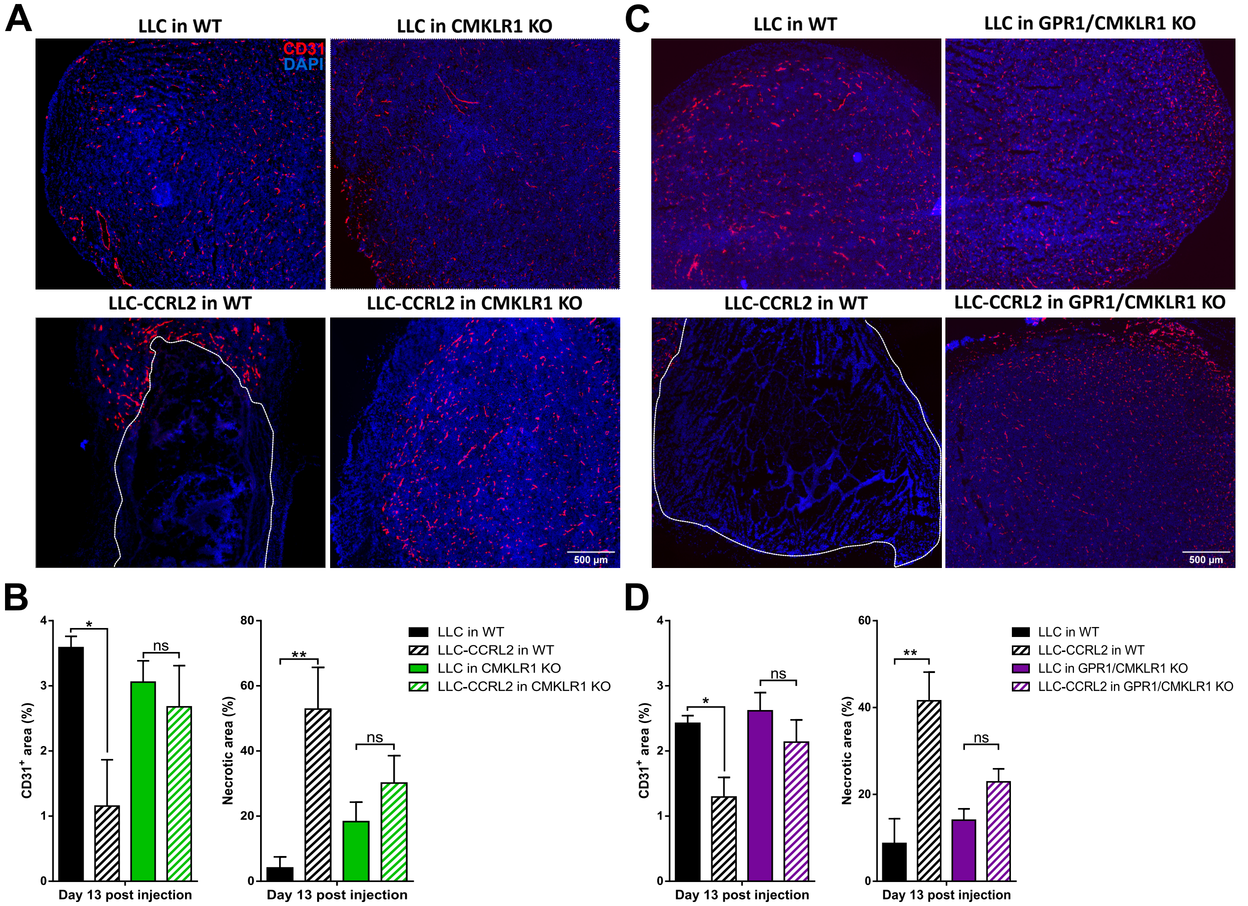

Figure S6. CCRL2 expression by LLC cells regulates neoangiogenesis. **(A)** Immunostaining of CD31 in LLC and LLC-CCRL2 tumors from WT and *Cmklr1* KO mice collected at day 13 post-injection. **(B)** Relative CD31^{+} and necrosis area in LLC and LLC-CCRL2 tumors collected from WT and *Cmklr1* KO mice at day 13. **(C)** Immunostaining of CD31 in LLC and LLC-CCRL2 tumors from WT and *Gpr1*/*Cmklr1* KO mice collected at day 13 post-injection. **(D)** Relative CD31^{+} and necrosis area in LLC and LLC-CCRL2 tumors collected from WT and *Gpr1*/*Cmklr1* KO mice at day 13. Scale bars = 500 µm. **P<0.01, *P<0.05, one-way ANOVA followed by Tukey-Kramer test for panels B and D.
